# Challenges and Perspectives for Vertical GaN-on-Si Trench MOS Reliability: From Leakage Current Analysis to Gate Stack Optimization

**DOI:** 10.3390/ma14092316

**Published:** 2021-04-29

**Authors:** Kalparupa Mukherjee, Carlo De Santi, Matteo Borga, Karen Geens, Shuzhen You, Benoit Bakeroot, Stefaan Decoutere, Patrick Diehle, Susanne Hübner, Frank Altmann, Matteo Buffolo, Gaudenzio Meneghesso, Enrico Zanoni, Matteo Meneghini

**Affiliations:** 1 Department of Information Engineering, University of Padua, 35131 Padova, Italy; desantic@dei.unipd.it (C.D.S.); matteo.buffolo@dei.unipd.it (M.B.); gauss@dei.unipd.it (G.M.); zanoni@dei.unipd.it (E.Z.); matteo.meneghini@unipd.it (M.M.); 2 Imec, Kapeldreef 75, 3001 Leuven, Belgium; matteo.borga@imec.be (M.B.); karen.geens@imec.be (K.G.); shuzhen.you@imec.be (S.Y.); stefaan.decoutere@imec.be (S.D.); 3 CMST Imec/UGent, 9052 Ghent, Belgium; Benoit.Bakeroot@UGent.be; 4 Fraunhofer Institute for Microstructure of Materials and Systems IMWS, Walter-Huelse-Strasse 1, 06120 Halle, Germany; patrick.diehle@imws.fraunhofer.de (P.D.); susanne.huebner@imws.fraunhofer.de (S.H.); frank.altmann@imws.fraunhofer.de (F.A.)

**Keywords:** vertical GaN, quasi-vertical GaN, reliability, trapping, degradation, MOS, trench MOS, threshold voltage

## Abstract

The vertical Gallium Nitride-on-Silicon (GaN-on-Si) trench metal-oxide-semiconductor field effect transistor (MOSFET) is a promising architecture for the development of efficient GaN-based power transistors on foreign substrates for power conversion applications. This work presents an overview of recent case studies, to discuss the most relevant challenges related to the development of reliable vertical GaN-on-Si trench MOSFETs. The focus lies on strategies to identify and tackle the most relevant reliability issues. First, we describe leakage and doping considerations, which must be considered to design vertical GaN-on-Si stacks with high breakdown voltage. Next, we describe gate design techniques to improve breakdown performance, through variation of dielectric composition coupled with optimization of the trench structure. Finally, we describe how to identify and compare trapping effects with the help of pulsed techniques, combined with light-assisted de-trapping analyses, in order to assess the dynamic performance of the devices.

## 1. Introduction

A central challenge of power electronics today is to address the continuously rising demands for safe and reliable control, conversion and distribution of energy, while maximizing the efficiency. Switched-mode power conversion strategies, with myriad applications [1,2,3,4,5], are now universally preferred over the simpler linear conversion methods due to the advantages of better flexibility, safety, and importantly, higher efficiency. The core requirement for efficient power conversion thus translates directly to highly efficient power transistors that can sustain repeated OFF/ON switching transitions with minimal switching and resistive losses. Higher operational frequencies are desirable, since they reduce the amount of energy transferred/cycle, which in turn reduces the size of the passive circuit components in the converters. Since higher frequencies will inevitably correspond to increased switching losses, the upper limit on the operational frequency (currently, in the MHz range) is majorly determined by the switching capabilities of the available power transistors. 

Silicon-based transistors have evolved over the years to meet the market needs; however further optimization is now bounded by the theoretical limits of Si. In this regard, wide-bandgap (WBG) semiconductors have found great consensus in being promising substitutes to Si transistors, derived from their superior figures of merit (FOMs). According to Baliga’s FOM (BFOM) (= εμEG3, VBR2Ron) [6], materials such as GaN and SiC present comprehensive improvements in the breakdown voltage (*V_BR_*) vs. on-resistance (*R_on_*) tradeoff. Comparing other FOMs provide easy estimations of the relevant metrics (a) conduction and switching losses from the on-resistance × output capacitance product ($R_{on}\times C_{oss}$) [7] and (b) power density from 1QgRonApackageRth [8] where $A_{package}$ is the package size and $R_{th}$ is the thermal resistance. The gate charge $Q_{g}$ represents the switching loss incurred by the charging and discharging cycles of the gate terminal. Here too, GaN emerges as the dominant choice over Si, as reviewed by Vecchia et al., in [1].

Thus, combining the improved transport, breakdown and thermal properties, the use of WBG materials enables cost and size-effective power transistors (converters) operating at high voltages and temperatures with higher speeds (lower switching losses), and with higher overall efficiency (lower conduction and switching losses).

Although GaN (BFOM = 3175 [9]) is superior to SiC (BFOM = 840 [9]) in most material properties, SiC has better thermal conductivity and is generally considered to be more relevant to the high voltage (>1200 V) application domain, while the commercial marketability of GaN is usually assumed to be in the low to mid voltage ≤650 V (power capability ≈ kW) domain [1,9,10]. This is primarily because of the current and voltage limitations [1,9,11] of the lateral configuration initially adopted for design of GaN power transistors. These devices were built to capitalize on the high-mobility high-density 2DEG formed at the AlGaN/GaN hetero-interface and indeed, several works on lateral GaN transistors have displayed impressive performances in the mid-voltage range [12,13,14], as a result of revolutionary improvements in GaN epitaxy and design over the last couple of decades.

However, to establish GaN power transistors as serious contenders in application markets such as Electric Vehicle/Hybrid Electric Vehicle (EV/HEV) [4] or power grids, voltage capabilities up to 1700–1800 V are required. To this aim, the research focus is now shifting to vertical GaN structures [2,3,15]. In addition to better heat management and normally off capabilities, vertical architectures overcome the breakdown voltage vs. device area tradeoff of lateral devices. With proper optimization, vertical transistors are also expected to present better reliability performance, since the electric field is moved within the bulk, eliminating surface issues.

Fully vertical GaN-on-GaN diode and transistor demonstrators have reported excellent performances (up to 3-4 kV capability [16,17,18,19,20,21,22,23,24]). However, GaN substrates are small and expensive, with wafer costs per unit area for GaN-on-GaN ranging up to $ 100/cm^2^ for 2-inch wafers [25,26]. Thus, currently these devices have limited commercial viability. Economically, the GaN-on-Si technology appears to be the most worthwhile for further development, with 8-inch wafers costing only $1 per unit area, potentially lowering wafer costs by 100 times. [25,26]. However, owing to the mismatches in lattice constant and thermal expansion coefficient between GaN and Si, the growth of thick GaN layers on Si are subject to high dislocation/defect densities, which makes the epitaxy especially challenging. Although some innovative techniques have been successful in fabricating fully vertical GaN-on-Si diodes [27,28,29,30,31], and a fully vertical GaN-on-Si power transistor (*V_BR_* = 520 V, *R_on_* = 5 mΩ.cm^2^) was recently demonstrated by Khadar et al. in [32] using substrate removal techniques, fully vertical GaN-on-Si technology is still in a very nascent stage. Recent results demonstrate the possibility of using engineered substrates (QST^®^), with a matched coefficient of thermal expansion, to enable low-cost vertical GaN FETs on large diameter wafers (8–12 inch) [33].

**Figure 1:**
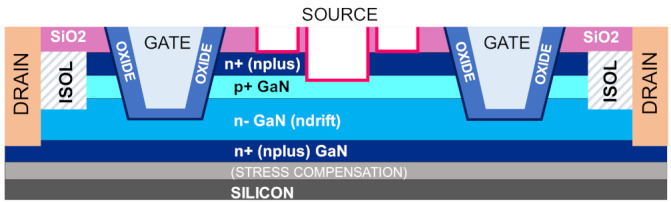
Schematic of a quasi-vertical n^+^-p^+^-n^−^-n^+^ GaN-on-Si trench MOS device.

For the development of the gate module and for the optimization of the drift region of vertical GaN devices, an important step is the development of quasi-vertical GaN-on-Si devices [3,27,34,35,36], based on the idea of maintaining the source and drain electrodes on the same side of the wafer. This approach allows us to understand, study and overcome the challenges related to the development of vertical GaN transistors, before moving to the full vertical layout. Quasi-vertical structures can build on the recent advancements into GaN-on-Si epitaxy achieved during research into lateral GaN devices, while providing better field management due to the vertical stack. Among the several available quasi-vertical configurations such as CAVETs [24,37], OG-FETs [38,39] or Fin FETs [22,40], the trench MOSFET [2,3,32,34,41,42,43,44,45,46] is a popular choice with high cell density. It is inherently a normally off device with low *R_on_*, and needs no regrowth of AlGaN/GaN channels. Figure 1 presents the schematic of a typical quasi-vertical GaN-on-Si trench MOSFET.

In the ON-state, the current in the quasi-vertical structure is sourced from the top n^+^ layer, and conducted vertically through the p^+^ GaN layer along the gate trench sidewalls. The current is then collected laterally through the bottom n^+^ layer, before being transported back to the surface through the drain metallization. A high doping of the n^+^ current-spreading layer ensures better current distribution, to minimize current crowding around the contact in the ON-state.

To design a reliable GaN-on-Si trench MOSFET, careful optimization of several interlinked physical parameters is required. As discussed earlier, the first design consideration, as for any power transistor, is to achieve a high *V_BR_* and low *R_on_* simultaneously. In this regard, the thickness and doping of the p-body and drift layer are the central constraints. The parameters need to be carefully engineered to ensure good reverse blocking capability in the OFF-state in addition to forward conduction in the ON-state. Regarding the M-O-S stack, gate design parameters such as dielectric composition and thickness are important in controlling the threshold voltage, leakage and gate capacitance of the device. The dielectric choice, in addition to structural optimization of the trench to minimize field crowding, controls the gate breakdown capability. Finally, the leakage and trapping needs to be minimized throughout the quasi-vertical stack.In this work, we will discuss recent case studies that address the impacts of different design choices on the performance of quasi-vertical trench MOSFETs, while demonstrating testing strategies used to identify and compare degradation mechanisms in such devices. In Section 2, p^+^-n^−^-n^+^ diode test structures are characterized; leakage modeling is used to identify the dominant mechanisms under reverse bias, and technology computer-aided design (TCAD) simulations are employed to compare the effects of high vs. low p-body doping, to present a trade-off useful for breakdown optimization. In Section 3, the optimization of the gate stack through the use of a bilayer dielectric is discussed. Specifically, the trapping and breakdown performance of bilayer (SiO_2_ + Al_2_O_3_) vs. unilayer (Al_2_O_3_) dielectrics are compared, and the effects of trench optimization are visualized by scanning electron microscopy (SEM) and transmission electron microscopy (TEM) analysis. In Section 4, methodologies for the assessment of the dynamic performance of the devices are presented. In addition, light-assisted experimental techniques are discussed, which improve the detection and understanding of trapping phenomena under low and high positive gate stresses.

## 2. OFF-State-Leakage and Doping Constraints of Quasi-Vertical GaN-on-Si Diodes from IMEC, Leuven, Belgium

In this section, we discuss the factors influencing the leakage current and the breakdown voltage of vertical GaN-on-Si stack, specifically designed for vertical trench-MOSFETs.

The growth of thick, mostly insulating GaN drift layers on Si was made possible during the last years thanks to the intense research on lateral power GaN devices; the main goal has been to improve the OFF-state blocking capability. For the move into vertical GaN devices, the drift layer modulation needs to be more rigorous, since in addition to sustaining high reverse biases in the OFF-state, it also needs to have a low resistivity in the ON-state. The ideal drift layer is thick, to sustain a large breakdown voltage, lightly doped, to ensure high mobility, thus allowing a good ON/OFF ratio, and has a low defect density, to minimize the defect-related leakage components [3]. Unintentionally doped drift layers are weakly n-type (10^16^ carriers/cm^3^ or above, [3,47,48]), due to residual impurities introduced during the growth process, such as silicon and oxygen [3,47,48,49,50,51,52].

In a vertical trench-MOSFET, the n^−^ drift region is in direct contact with the p-body, that may have Mg concentrations in excess of 10^18^–10^19^ cm^−3^. To optimize the breakdown voltage of vertical power FETs, it is therefore important to minimize both the leakage through the drift region, and to ensure that the p-body/drift region junction can sustain the high vertical field when the device is in the OFF-state [3,31,53,54,55].

Magnesium doping in GaN has been reported to form acceptor states located 0.16 eV above the valence band [56,57]. This relatively deep energy level results in incomplete thermal ionization of Mg acceptors at room temperature. Since the presence of hydrogen during MOCVD growth of p-type GaN can passivate the Mg-dopant through the formation of Mg-H bonds [58], a post-growth annealing treatment (while ensuring energies are lower than the threshold to create native defects) is necessary to ensure a high conductivity and hole density.

There are several possible leakage paths in the OFF-state [53,59,60]. In the quasi-vertical layout, parasitic leakage along the etch sidewalls and the bulk regions might be dominant and needs to be minimized. Other leakage paths may be present along the passivation layers, or vertically along the entire stack, reaching the substrate. To minimize the vertical leakage, the prevalent leakage mechanisms among different technology variations need to be understood, to enable directed improvements.

In performing leakage analysis of reverse biased p^+^n diodes, the conduction mechanisms through dielectrics subjected to high electric fields have been found to be applicable [53,59,61]. For low to medium reverse bias, the relevant mechanisms are usually electrode-limited related to the quality of the metal-semiconductor contacts. However, these mechanisms are usually not relevant in good vertical designs. As such, bulk conduction mechanisms are more relevant, in particular, variable range hopping (VRH) [54,55,59,62,63,64,65,66,67,68,69,70,71], Poole-Frenkel emission [59,63,64,65,66,67,68,69,72,73,74,75,76,77,78], and space charge limited conduction (SCLC) [31,47,53,79].

**Figure 2:**
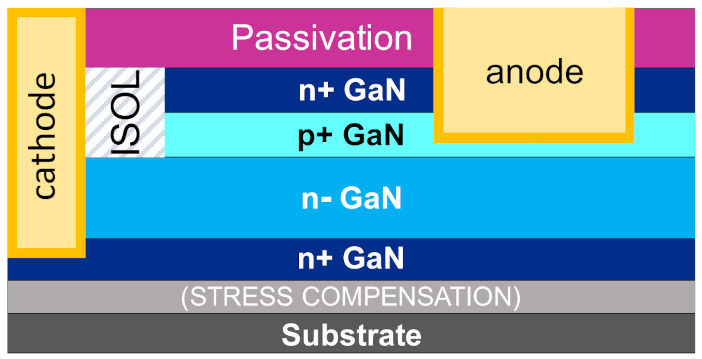
Schematic of the quasi-vertical p^+^-n GaN-on-Si diodes.

For investigating the doping and leakage issues under OFF-state within the vertical stack, it is useful to consider the simpler quasi-vertical diode structures, which form the fundamental block of the full MOSFET. The test vehicles used for the following study were aimed at understanding the p^+^-n^−^-n^+^ stack; the schematic is presented in Figure 2. Fabricated on a 200 mm Si substrate, the diodes have Mg doping with N_A_ = 6 × 10^19^ cm^−3^ within the p^+^ layer, and a weakly n-type drift layer with n = 4 × 10^16^ cm^−3^. The cathode is at the buried n^+^ layer below the n drift region. The reverse breakdown voltage was measured to be 170 V on these specific structures, having a drift layer thickness equal to 750 nm [45,55].

### 2.1. Leakage Modeling

**Figure 3:**
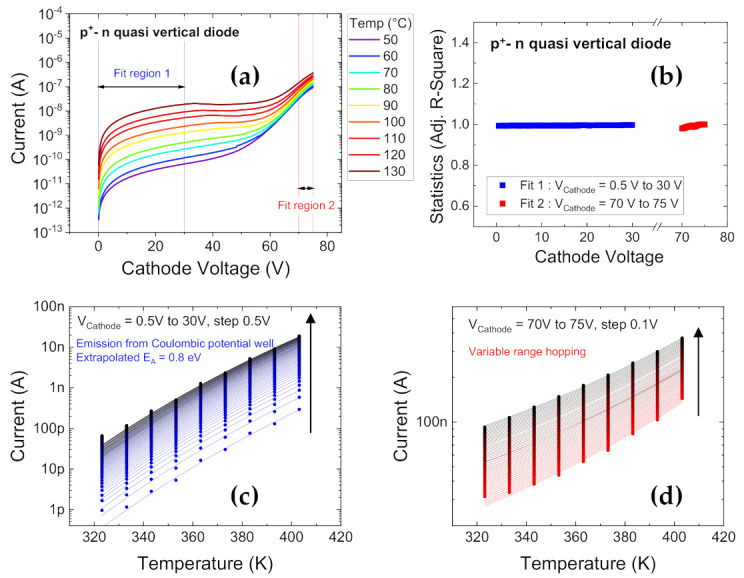
Modeling of the reverse-biased characteristics of the p^+^-n diodes under test [55]. (**a**) Reverse diode characteristics from T = 50 °C to 130 °C. The two distinct regions identified in (**a**) are fitted using the Coulombic potential well model in (**c**) for V_Cathode_ from 0.5 V to 30 V (in direction of arrow), and using the variable range hopping model in (**d**) for V_Cathode_ from 70 V to 75 V (in direction of arrow). (**b**) Displays the good conformity of the fits with adjusted R^2^ ≈1 using the statistical parameter of adjusted R-square (coefficient of determination).

Since individual leakage mechanisms have distinct temperature dependencies, temperature dependent I-V behavior is obtained. Reverse biased diode characteristics over a range of temperatures (T) from 50 °C to 130 °C are displayed in Figure 3a. The maximum cathode voltage (V_Cathode_) was limited to *V_BR_*/2 to avoid degrading the samples, and obtain clean trends with T for medium voltages. The presence of two different natures of variation with T is found, hence two regions were identified to be modelled separately.

The first region, from V_Cathode_ = 0 V to 30 V, with a strong increase in current with temperature, was found to best represent conduction from Coulombic traps through thermionic emission [54,72]. The corresponding fit data is presented in Figure 3c. This mechanism is based on the assumption that the potential around traps at low electric fields can be considered Coulombic, while at higher fields, according to the Poole-Frenkel effect, a lowering of the potential barrier is expected with a square root dependency on field, strengthening the emission process of the trap [59,72,73,74]. This is expressed in the following formula, and the parameters are defined in [55]:

(1) ITE=AT2exp−EAkT,

(2) en ∝ exp−ET−βF12kBT,

(3) β=q3πε,

The slope extracted from the fitting (not shown) revealed an activation energy *E_A_* of ≈0.85 eV, usually associated with the presence of carbon acceptors [80,81], with an effective lowering in *E_A_* (Δ*E_A_*) = 70 meV, the corresponding Poole-Frenkel coefficient *β* (=1.77 × 10^−5^ eV V^−1/2^ m^1/2^) was found to be close to the theoretical value [55]

The second region, from V_Cathode_ = 70 V to 75 V, was modelled using variable range hopping (VRH), the leakage evolution fit to the VRH model is presented in Figure 3d. The corresponding equation is written as in Equation (4), and the parameters are described in [55]:

(4) IVRH=I0exp−1.76T0T14+CVRHT0T34F2

VRH describes the conduction of electrons across multiple trap states distributed within the bandgap. With the high occurrence of substantial defect densities in GaN epitaxial layers, VRH is commonly observed in GaN diodes [59,62,63,64,65,66,67,68,69,70], ascribed to the hopping of charged carriers through localized defect states in depletion regions.

For both the fits in Figure 3c,d, the adjusted R-Square (Adj. R-Square) [82] is found to be close to 1, as presented in Figure 3b, attesting to the good conformity of the fits. The R-square, also referred to as the coefficient of determination, always lies between 0 to 1, corresponding to whether the fit line is able to describe 0% or 100% of the variability of the data around the mean. Adj. R-Square is a modification which takes the number of predictors (within the fitted line) into account.

### 2.2. Simulation of Doping Constraints in Diode Breakdown

The investigation of breakdown issues is especially suited to using TCAD simulations, which provide versatile, non-destructive and rapid optimization solutions. A representative and simplified (fully vertical) model of the test devices was built using the Sentaurus tool from Synopsys in order to investigate the nature of breakdown, relative to the chosen concentration of p-doping in GaN diodes [55]. The drift diffusion transport model is used, along with appropriate polarization, mobility and recombination models. The n^+^ layers are doped with N_D_ = 5 × 10^18^ cm^−3^, and the n^−^ drift layer doping is fixed at N_D_ = 4 × 10^16^ cm^−3^. For the p-body doping, Mg is defined as the dopant species. As discussed earlier, the Mg acceptors are not expected to be completely ionized at room temperature. Hence, to correctly estimate the effects of p-doping, using the incomplete ionization model is more physical. This model takes the parameters of the individual acceptor species into account, in particular, the ionization energy. Based on this, the simulator internally computes the effective doping concentration under different conditions. For example, a defined Mg concentration of N_A_ = 6 × 10^19^ cm^−3^ with an ionization energy of 0.16 eV, leads to an effective base doping within the p-GaN region of ≈ 4 × 10^18^ cm^−3^ (6%), except within the depletion regions around the p-n junctions, where the defined N_A_ is almost completely ionized.

**Figure 4:**
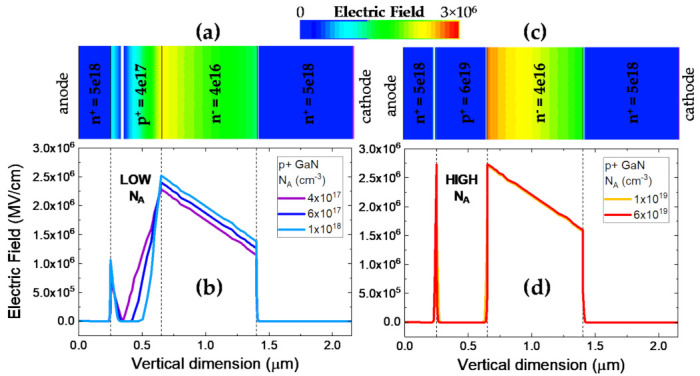
TCAD modeling of vertical p^+^-n diodes under different p-doping conditions describing the expected breakdown processes (**a**) TCAD structure visualized at N_A_ = 4 × 10^17^ cm^−3^; (**b**) Electric field evolution for low p doping values illustrates complete depletion (punch-through) of the p-GaN region; (**c**) TCAD structure visualized at N_A_ = 6 × 10^19^ cm^−3^; (**d**) Electric field evolution for high p doping values illustrates high electric fields (approaching critical field for GaN) at the p^+^-n^−^ interface.

Since the measured breakdown voltage of the test diodes is 170 V, the electric field evolution within the vertical diode is visualized at 160 V with different N_A_ values in Figure 4. In Figure 4a,b, the chosen N_A_ values are relatively low = 4 × 10^17^ cm^−3^ (see Figure 4a), 6 × 10^17^ cm^−3^ and 1 × 10^18^ cm^−3^. In this scenario, the p-GaN region is observed to be severely depleted, with reach through occurring for the N_A_ = 4 × 10^17^ cm^−3^ case, once the depletion regions from the n^+^-p and p-n^−^ junctions intersect. Thus, a lower bound for setting the p-doping is identified owing to this constraint. In a real growth scenario, this constraint could be considerably tighter. If the reduction in Mg concentrations due to hydrogen passivation or other impurities were considered, the breakdown could occur faster (at lower voltages) for equivalent N_A_ settings.

In Figure 4c,d the higher N_A_ values are considered, including the representative value for the structures under test with N_A_ = 6 × 10^19^ cm^−3^ (see Figure 4c). For these cases, the applied voltage drops almost entirely across the lightly doped n^−^ GaN region, leading to smaller depletion of the p^+^ GaN layer. On the other hand, the peak electric field at the p^+^ to n^−^ interface is significantly higher. In this scenario, breakdown in expected to be field-triggered, in fact, for N_A_ = 6 × 10^19^ cm^−3^, we are approaching critical field for GaN (≈ 3 MV/cm [83]) at the 160 V condition, which is found to agree reasonably well with the measured breakdown voltage of 170 V. Thus, the higher bound for N_A_ settings is identified.

Based on the results in Section 2, we infer that the density of defects within the drift region need to be optimized to control the leakage current and its temperature sensitivity. The contribution of the residual carbon concentration is found to be relevant to the low voltage regimes, and needs to be optimized to improve the leakage performance. Regarding p-doping-induced constraints on the breakdown voltage, for a lightly doped drift layer, keeping the p-doping low can reduce the peak electric field, pushing *V_BR_* to higher voltages. However, the trade-off dictates that the value still needs to be high enough to avoid complete depletion of the p GaN layer unexpectedly at low voltages.

## 3. OFF-State and ON-State–Optimization of the M-O-S Stack in Quasi-Vertical MOSFETs from IMEC, Leuven, Belgium

This section describes recent results on the degradation and optimization of the MOS gate stack used for GaN-on-Si vertical MOSFETs.

The reliability of the gate stack is highly influenced by the choice of the oxide in trench MOSFETs, since the insulator is vulnerable to repeated stressing during the operation of the power devices over time [46,84,85]. Specifically, the properties of the insulator can greatly affect the leakage, breakdown and trapping performance of the M-O-S stack under positive gate stresses. One of the essential requirements for a gate oxide is to have high band offsets with GaN, which is critical to limit the leakage current [86,87,88]. In this regard, while materials such as silicon nitride or hafnium oxide (band offsets around 1 eV) are less favored, Al_2_O_3_ [89,90] and SiO_2_ [32,34] have emerged as popular choices with conduction band offsets (ΔE_C_) of 2.1 and 2.5 eV, respectively. Al_2_O_3_ presents good metrics [86,87,88]: in addition to having a high bandgap (8.9 eV), high k (dielectric constant = 9.0), and reasonably high breakdown strength (~10 MV/cm), improvements in deposition techniques now allow Al_2_O_3_/GaN interfaces to be formed with very low interface state densities [88,91,92]. SiO_2_ also has a high bandgap (9.1 eV), and its advantage is high chemical stability, which extends to high operational stability in the devices.

Since the reliability of the MOS framework is still not completely understood, there has been limited effort in exploring alternatives to the conventional MOS structure with an unilayer dielectric. In particular, the approach of using bilayer dielectrics (with a thin interface dielectric followed by a thicker insulator), which has been found to be advantageous for Si MOSFET design, could potentially be very valuable for GaN-based MOSFETs as well. However, inherent reliability risks could be worsened with increasing complexity in the dielectric deposition process. To truly capitalize on the effects of improved dielectrics, the bulk GaN etch process, in particular the formation of the trench itself, needs to be highly optimized. The shape of the trench is usually optimized [93,94,95,96,97] to find the best combination of *V_BR_* and *R_on_*; deep trenches with rounded corners have been reported to display good metrics [93,98,99]. However, for higher trench depths (over-etch) extending beyond the p-body, the peak field under the OFF-state could be aggravated [93]. The overall etching process is aimed at creating smooth sidewalls, and preventing irregularities such as pits or voids, especially at the bottom trench corners where the peak fields are expected [93,94,95,96,97].

### 3.1. Optimising Dielectric Composition

**Figure 5:**
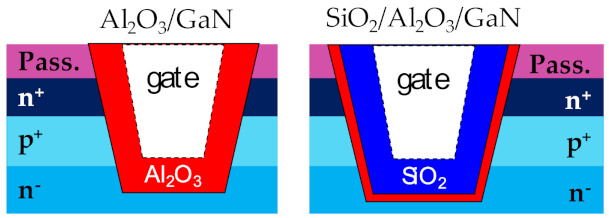
Dielectric composition of the devices under test. The first configuration is an unilayer of 35 nm Al_2_O_3_ at the GaN interface, while the second has a bilayer composition: 35 nm of SiO_2_, then 2.5 nm of Al_2_O_3_ at the GaN interface.

This section demonstrates the advantages of employing a bilayer insulator composition in quasi-vertical MOSFETs through DC and pulsed measurements, and TCAD simulations [46]. The devices under test are GaN-on-Si trench MOSFETs, structurally similar to Figure 1. During Atomic Layer Etch (ALE) processing steps, an O_2_ plasma is used to oxidize the GaN after which a BCl3 dry etch step is executed to remove the oxidized GaN layer. The amount of ALE cycles has been optimized to ensure a good profile of the gate trench, removing in total ~25 nm. In this section, we discuss the effects of the dielectric composition around the gate trench, as illustrated in Figure 5. The Al_2_O_3_ deposition is performed using atomic layer deposition (ALD) at 300 °C, while the SiO_2_ in the bi-layer is deposited using plasma-enhanced chemical vapor deposition (PECVD) at a deposition temperature of 400 °C. The focal idea was to compare the robustness of devices fabricated with a bilayer dielectric composed of SiO_2_ and Al_2_O_3_ to devices with a traditional unilayer dielectric of Al_2_O_3_. Effectively, the bilayer stack should combine the merits of SiO_2_ as a bulk insulator with the ability of Al_2_O_3_ to create a high-quality interface to GaN.

As expected, the gate-source and gate-drain diode leakage of the bilayer devices was found to be lower by a couple of orders of magnitude [46]. This is attributed to the intrinsically higher breakdown field of SiO_2_, as well as the additional barrier (conduction band discontinuity at the Al_2_O_3_/SiO_2_ interface of 0.4 eV [86]) to thermionic leakage from the channel to the gate, introduced by the bilayer configuration.

To evaluate the reliability of the two stacks under the ON-state, forward gate breakdown step stress tests were performed, where the gate voltage was incremented from 0 V in steps of 3V, while V_DS_ was constant at 1 V. Very little dispersion in breakdown voltage was observed across several devices, and the gate breakdown voltage for the unilayer and bilayer configurations were found to be 9 V and 27 V [46], the bilayer devices displaying an improvement of three times.

**Figure 6:**
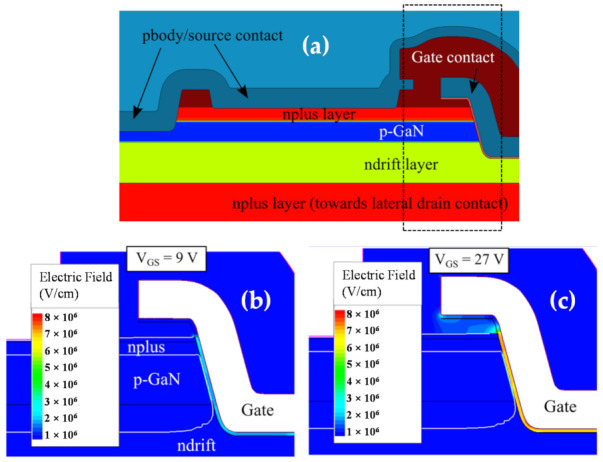
(**a**) Schematic of the simulated quasi-vertical trench MOSFET. Electric field distribution around the trench edges at the measured ON-state breakdown voltage visualized for (**b**) unilayer: Al_2_O_3_/GaN devices and (**c**) bilayer: SiO_2_/Al_2_O_3_/GaN devices.

In Figure 6, the schematic of the simulated device (Figure 6a), and the electric field distribution within the unilayer and bilayer oxides are visualized at their respective gate breakdown voltages.

In the ON-state, the channel exists continuously along the trench sidewalls. Thus, the applied gate voltage falls entirely within the oxide layer, and the internal field grows rapidly, as illustrated in Figure 6b,c. This condition can then be used to estimate the critical electric field for the two gate dielectric compositions. From theoretical considerations, the unilayer Al_2_O_3_ devices are expected to have an average critical electric field value of 2.6 MV/cm (9 V/35 nm), while the bilayer devices are estimated to have a critical electric field value of 7.5 MV/cm (26.2 V/35 nm) for the SiO_2_ layer, and 3.2 MV/cm (0.80 V/2.5 nm) for the Al_2_O_3_ layer [46]. These values are well substantiated by the simulated electric fields in Figure 6 obtained at the respective breakdown voltages.

**Figure 7:**
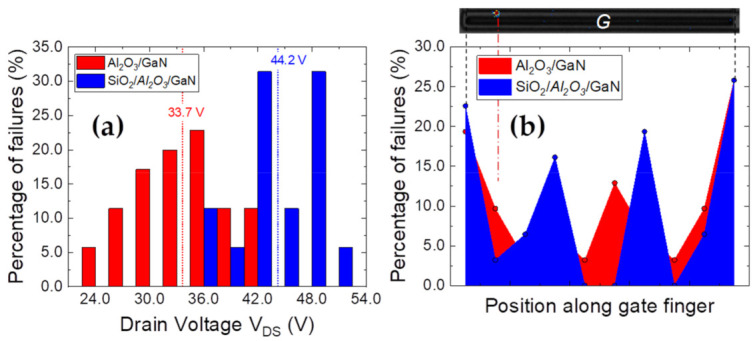
OFF-state drain step stress performance at V_GS_ = 0 V for a 35-device sample set (**a**) Comparison of the experimental breakdown values for both unilayer and bilayer cases (**b**) Localization of the failure spots along the gate finger, collected from observed EL spots (an example of an EL spot shown for reference at top) at corresponding *V_BR_* values.

The second set of measurements were aimed at comparing OFF-state performance of the dielectric stacks. Figure 7 presents the results of drain step stress until breakdown, coupled with electroluminescence (EL) studies, performed at V_GS_ =0 V on 35 devices from each wafer. During each stress step, an EL image was simultaneously generated with an acquisition time of 40 s [46]. In the OFF-state, the applied stress voltage is distributed across the depleted drift layer, in addition to the dielectric stack, resulting in correspondingly higher breakdown voltages for both unilayer and bilayer devices. The *V_BR_* distribution for the tested devices is compared in Figure 7a, wherein the bilayer emerges as clearly superior, with an average *V_BR_* improvement of 10 V.

An example of an EL spot observed along the gate finger at *V_BR_*, reflecting the region of breakdown in the devices, is shown in Figure 7b, along with a collated map of the breakdown spots for all tested devices, identified through EL acquisitions obtained during the step stress process, and on reaching failure. The results clearly indicate a preferential failure occurrence at the corners of the gate fingers, independent of the dielectric deposition.

The measurements displayed in Figure 7 were performed using microprobes fitted with an optimized current limiting circuit, in order to protect the failed devices from thermal runaway, and to preserve them for further post-failure analyses by TEM and Energy Dispersive X-ray Spectroscopy (EDX) [100,101] to identify the cause of breakdown [102].

**Figure 8:**
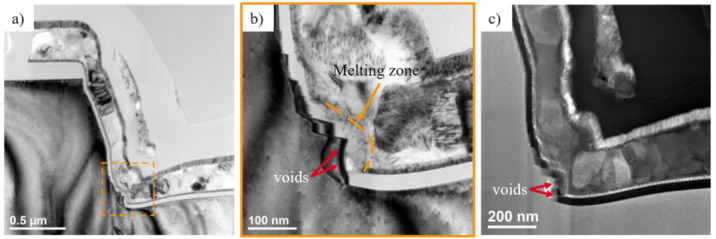
TEM analysis of defect at gate trench of a bilayer device at the position of an EL spot (**a**,**b**) BF-TEM and (**c**) ADF-STEM images of an approx. 50 nm thin lamella.

Compared to the size of the original defect, an observed EL spot represents a relatively wide area in which the original defect could be present. Screening is necessary to precisely localize the defect within the observed EL spot area, which can be done by performing alternating focused ion beam (FIB) milling and SEM imaging [100]. After screening of the defect, TEM investigations were performed at various lamella thicknesses starting from 1.5 µm down to 50 nm to search for of a particular defect. Figure 8 exemplary displays the results of a defect analysis of a stressed bilayer device at the location of a particular EL spot, with a focus on the gate trench corners.

Device failure was identified to have been caused by an electrical breakdown of the gate isolation at the bottom edges of the trench, and was correlated with the presence of several abrupt steps of the gate trench sidewall [102]. While the defect structure was found to coincide with a melted area and several voids (see Figure 8b,c) as a consequence of gate shorts [102], EDX analysis on failed devices (not shown here, but reported in [102]) revealed that the breakdown of the gate isolation resulted in minor migrations of silicon and oxygen, and a dominant migration of nitrogen into the gate oxide.

**Figure 9:**
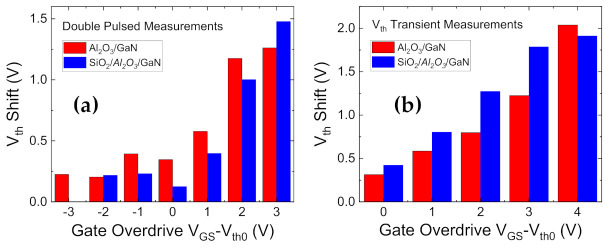
Comparison of bilayer vs. unilayer V_th_ shifts relative to the unstressed threshold voltage using (**a**). Double pulsed characteristics and (**b**) V_th_ transient tests.

To complete the investigation into the relative merits/demerits of the bilayer composition, trapping analyses using double pulsed [44,103] and on-the-fly transient [44,104,105] measurements were performed on several devices from both wafers, as presented in Figure 9. More details on the test methods will be provided in Section 4. The shift in the threshold voltage (ΔV_th_) is compared for identical positive gate overdrive stresses.

The V_th_ shifts are comparable or slightly higher for the bilayer case, which could be due to additional trapping sites generated at the additional interface within the dielectric. However, the trapping performance for both the compositions is primarily comparable, which implies that most of the trapping can be presumed to occur at the interface and/or border traps near the shared GaN/Al_2_O_3_ region [44,106,107].

### 3.2. Optimising Trench Fabrication

In Section 3.1, the cause of breakdown was correlated to non-idealities around the trench edges. In this section, the cross-sectional analyses to identify the underlying issue, and to visualize improvements in the gate trench etch process, are summarized [102], in an effort to understand how to improve breakdown performance.

The investigated devices are GaN-on-Si trench MOSFETs with bilayer gate dielectric compositions. The fabrication process of the gate trench involved a bulk GaN etch process followed by an ALE and wet cleaning process. The first set of devices (Wafer A) are from the bilayer wafer presented in Section 3.1 (see Figure 8). The second set of devices (Wafer B) are taken from a wafer with an optimized ALE processing and wet cleaning sequence.

**Figure 10:**
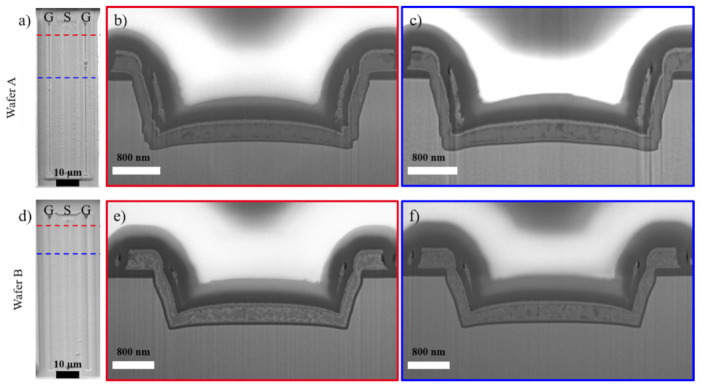
Slice and View analysis by FIB-SEM along the gate finger of devices from (**a**–**c**) Wafer A and from (**d**–**f**) Wafer B. (**a**) and (**d**) SEM top view images of the devices. The positions of the cross sections are marked by colored, dashed lines. (**b**,**c**) and (**e**,**f**) SEM cross sectional images. The colored frames correspond to the colored dashed lines in (**a**) and (**d**).

During the initial FIB-SEM investigation to isolate the defective/shorted gate, irregularities of the trench structure of Wafer A were observed. Hence, slice-and-view FIB-SEM analysis [100,101,108,109] was undertaken to study the trench at different locations along the gate finger, as presented in Figure 10.

Several steep steps of varying shape and length were observed at each cross section along the trench sidewalls, dominantly at the lower trench corners. Since these irregularities are associated with accelerated degradations, drawing from these observations, the ALE and wet cleaning processes were improved during the fabrication of Wafer B. As displayed in Figure 10d–f, the newly fabricated trench gates have clean sidewalls, with no observed roughness or steps. Further TEM analysis [102] also corroborated these observations.

From the results in Section 3, we can improve the general understanding of the degradation mechanisms that occur within the gate stack, when subjected to prolonged gate and drain stresses. Bilayer dielectric compositions, utilizing the good interface properties of Al_2_O_3_ to GaN and the improved stability of the SiO_2_ material, were found to be highly advantageous to breakdown performance of GaN trench MOSFETs, without significant worsening of trapping effects. However, before improving other design parameters, the fundamental GaN etch process must be robust. Microstructural defects formed during fabrication of the gate trench sidewalls can manifest in worsened reliability and faster breakdown, hence optimization techniques to minimize etch roughness are critical.

## 4. ON-State-Light Assisted Analysis of Trapping Mechanisms in Quasi-Vertical MOSFETs from IMEC, Leuven, Belgium

For reliable ON-state operation of GaN MOSFETs, it is fundamental to understand and minimize the trapping states for the insulator/GaN interface. Since III-V semiconductors have no native oxides, developing high quality oxide films on GaN is difficult. The progress in the application of the atomic layer deposition technique has allowed the successful deposition of low-defect Al_2_O_3_ films on GaN, improving the performances of MOS structures. However, identifying relevant trapping sites and the induced threshold voltage V_th_ instabilities [44,89,106,107,110,111] due to limited controllability of the GaN surface potential continues to be a primary task to the adoption of GaN vertical MOSFETs in real applications.

In Section 3.1, the trap impacts on threshold voltage were found to be comparable between bilayer and unilayer dielectric cases, indicating that states at or near the GaN/Al_2_O_3_ interface are presumably the major contributing factor to bias threshold instability (BTI) observations.

In this section, we focus on unilayer Al_2_O_3_-only trench MOSFET devices with an average V_th_ of 2 V, with device structure similar to Figure 1, to understand the trapping mechanisms through characterization of induced V_th_ shifts [44]. Within the Al_2_O_3_/GaN system, three fundamental trapping locations have been identified [106,107]. Trap states within the bulk dielectric and near-interface or border sites depend strongly on the properties of the deposited Al_2_O_3_, while the states along the Al_2_O_3_/GaN interface (quantified by the interface state density D_it_) correlate to the quality of the dielectric/semiconductor boundary, and of the process. For a wide band-gap material such as GaN, it is often difficult to isolate the effects of energetically deep trap states. This is where light energy, and especially the application of UV light with energies approaching/higher than the GaN band-gap, is valuable. In the following results, we investigated V_th_ shifts under positive gate stress, by combining analytical techniques to identify trap processes and associated recovery dynamics. In each case, light energy is used to support the analyses, and provide further insight into the physical origin of the trap states.

**Figure 11:**
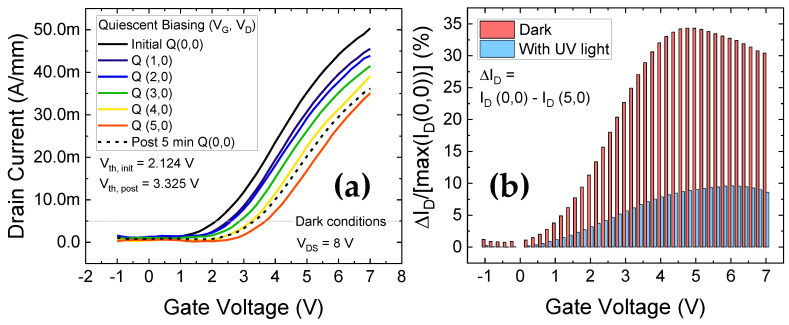
Double pulsed characteristics; (**a**) Measurements under dark conditions show a ΔV_th_ = 1.2 V and very little recovery in the measured I_D_V_G_, 5 min after the stress at Q (5,0); (**b**) Comparison of current level shifts measured under no light and UV light. Under UV illumination, shifts are lower under during stress conditions, and post-stress recovery is faster.

The first set of measurements to test the dynamic performance of the devices, as summarized in Figure 11, are double pulsed measurements. The double pulse measurement system is a powerful high voltage, high speed setup to analyze the dynamic performance of devices by synchronously pulsing the gate and drain voltages. The pulsing setup switches between the quiescent (stress conditions) and measurement phases within relatively short time scales (μs). The V_G_ stress settings are incremented from V_G,Stress_ = 0 V to 5 V, V_D,Stress_ = 0 V for a quiescent time of t_Q_ = 100 μs, and the I_D_-V_G_ measurement settings were V_GS_ = −1 to 7 V, V_DS_ = 8 V for a measurement time t_meas_ = 1 μs. In Figure 11a, the measurements were performed in dark conditions, displaying a positive shift in V_th_ (PBTI) of 1.2 V for Q (5,0) (V_th_ calculated as the voltage intercept at I_D_ = 5 mA/mm). The V_th_ shift can be attributed to the fast-pulsed stressing configuration, with no recovery intervals between the progressively stronger stress conditions.

After a rest period of 5 min following the positive gate stress at Q (5,0), the I_D_-V_G_ measured for Q (0,0) condition still showed substantial degradation from the initial I_D_-V_G_ characteristic at Q (0,0), indicating semi-permanent trapping processes. This can also be visualized by plotting the ΔI_D_/I_D, max_ ratio in Figure 11b for the high stress Q (5,0) condition.

The shift in the current levels under stress was 30% of the pre-stressed current maximum, while 5 min of recovery reduced it to 25–27%. On the other hand, repeating the same stress-recovery cycles as in Figure 11a, but under the presence of UV light displayed substantial improvement. As highlighted in Figure 11b, under UV light, for the highest stress condition of Q (5,0), the shift in the current levels was less than 10%. Furthermore, letting the device recover for 5 min thereafter, the deviation in the I_D_-V_G_ at Q (0,0) from the unstressed initial I_D_-V_G_ at Q (0,0) was found to be negligible (ΔI_D_/I_D, max_ ≈ 2–3%, not shown).

Based on these observations, a powerful transient setup was employed to take a closer look at the evolution of induced V_th_ shifts under longer gate stress durations, in the presence of different monochromatic light energies. This versatile setup accurately evaluates V_th_ transients in the 10 μs–100 s range where a typical measurement consists of 100 s of stress and 100 s of recovery. Twenty-two fast I_D_–V_G_ measurements of 10 μs each are performed during the stress/recovery phases to compare the evolution of V_th_. During initial measurements using this technique, small negative V_th_ shifts were observed at low stress voltages [89,112], and high positive V_th_ shifts were observed for gate stresses of 4 V and higher [44]. To investigate the effects of light-assisted de-trapping, the recovery was repeated under different wavelengths of light, following 100 s of trap filling at V_G,Stress_ = 5 V, and V_D,Stress_= 0 V.

**Figure 12:**
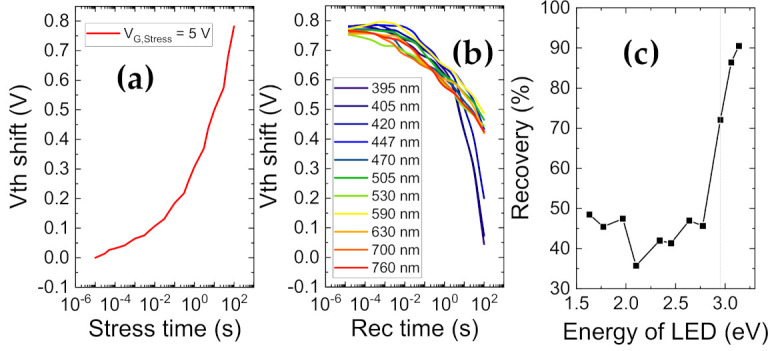
V_th_ transient measurements (**a**) Shift in V_th_ (V_th_−V_th@10 μs_) during stress phase of 100 s at V_G,Stress_ = 5 V. (**b**) V_th_ evolution during recovery phase of 100 s at V_G,Stress_ = 0 V under varying light wavelengths from 760 nm to 395 nm, following equivalent stress phases as described in (**a**), (**c**) absolute V_th_ shift during recovery (V_th@100s_−V_th@10μs_ during recovery) versus the light energy.

Figure 12 presents the results of the light-assisted V_th_ transient technique. In Figure 12a, a positive V_th_ shift of 0.75 V is seen after 100 s of stress at V_G,Stress_ = 5 V. The recovery transient (at V_G,Rec_ = 0 V and V_D,Rec_ = 0 V) in response to this stress, was measured under dark and under monochromatic light energies from 1.6 eV to 3.1 eV, as illustrated in Figure 12b,c. Under dark conditions, the recovery is slow and hence incomplete [113] at the end of the 100 s of recovery phase. For low photon energies, such as 760 nm, only 50% (0.35 V) of the stress-induced PBTI was recoverable within 100 s. For higher photon energies, de-trapping was found to be gradually accelerated. The threshold energy (associated to the lowest energetic position of deep bulk states) for improved de-trapping was identified to be 2.95 eV (420 nm), while complete recovery of the 0.75 V of positive V_th_ shift was observed within the 100 s window for the 3.1 eV (395 nm) case. As can be noticed in Figure 12b, all photon energies below 2.7 eV did not induce any significant changes, with small/negligible recovery. Small variations observed below this threshold in Figure 12c may be ascribed to small (5–10%) measurement inconsistencies and/or noise. 

A direct takeaway from this would be the presence of trap states located energetically between 2.9 eV and 3.1 eV from the conduction band of the oxide, which equates to 0.8 to 1.0 eV from the conduction band of the semiconductor, considering a conduction band offset of 2.16 eV [86] at the Al_2_O_3_/GaN interface.

**Figure 13:**
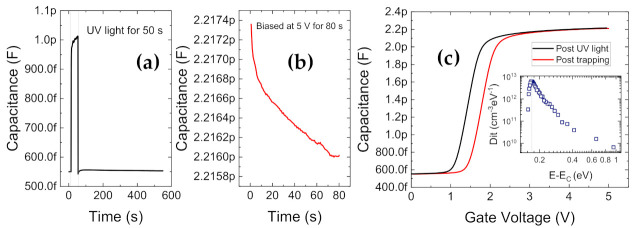
Photoassisted CV method for D_it_ extraction; (**a**) Capacitance-time transient during exposure to UV light at V_G_ = 0 V; (**b**) Capacitance-time transient during filling of traps at V_G_ = 5 V. (**c**) C-V comparison between detrapped (after UV light) and trapped state. (inset) Electron D_it_ vs. E_G._

The final light-assisted technique to identify trap distributions is the photo assisted CV method [44,114]. This measurement approach evaluates the distribution of interface states located along the gate dielectric interface to GaN. In this method, capacitance-voltage measurements, obtained under a photo-assisted de-trapped condition and a bias-induced trapped condition, are compared to quantify the interface state density. The use of UV light allows us to empty all defects at the interface (when the device is in depletion) to probe interface states deep within the bandgap. The results of the photo-assisted CV experiment are displayed in Figure 13.

The devices are biased in depletion condition for a short time and then exposed to UV light in order to empty all traps at the interface, as shown in Figure 13a. In the presence of UV light, electron-hole pairs are generated, accompanied by an increasing capacitance transient due to the release of trapped charge inside the depleted region. The duration of UV exposure is 50 s, until the capacitance level saturates. This is followed by a longer time interval in the dark (500 s) to allow enough time for the excess photo-generated carriers to leave the system and reach thermal equilibrium. Then, the de-trapped capacitance-voltage curve from depletion to accumulation is measured from V_G_ = 0 V to 5 V (see Figure 13c). Bias at the end voltage (5 V) is maintained for a moderate filling time (80 s), to induce charge trapping at insulator and interface states, as shown in Figure 13b. Finally, the second C-V curve of the trapped device is measured from accumulation to depletion. The difference in C-V slope of the trapped and de-trapped curves allows the extraction of D_it_ versus energy, while the fixed shift in the curves is proportional to the amount of charge trapped in the bulk of the oxide and/or in near-interfacial or border traps. The D_it_ profile (inset of Figure 13c) reveals shallow traps located around 0.3 eV from the conduction band.

**Figure 14:**
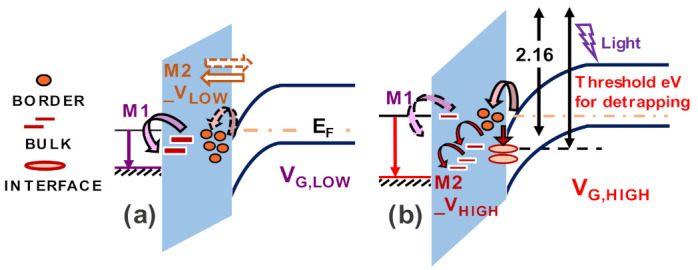
Energy band diagrams illustrating trapping locations in the Metal/Al_2_O_3_/GaN system (**a**) mechanisms activated at low V_G_ stress. M1: negative ΔV_th_ due to detrapped electrons from oxide towards metal. M2_V_LOW_: moderate and recoverable positive ΔV_th_ due to injection of electrons from GaN accumulation into the border oxide traps; (**b**) mechanisms strengthened at high gate stress, M2_V_HIGH_: strong positive ΔV_th_ due to electrons injection into energetically deeper interface traps or bulk states in the dielectric. M2_V_HIGH_ causes semi-permanent trapping which requires external light energy (inducing de-trapping) for achieving fast recovery of V_th_ [44].

Based on the observations in Section 4, the following inferences regarding relevant trapping mechanisms under forward gate stress can be drawn, as also summarized in Figure 14.

The small NBTI observed during V_th_ transients at low gate stresses (≤2 V) is attributed to de-trapping of electrons within the gate oxide to the metal (M1 in Figure 14). When medium gate stresses are applied (≈3–4 V), small amounts of PBTI can be attributed to electron trapping from the semiconductor towards border states in the dielectric (M2_V_LOW_ in Figure 14). V_th_ shifts owing to this process are recoverable once stress is removed and the Fermi level is restored, even under dark conditions if enough recovery time is provided. For high gate stresses (≥4V), strong PBTI is induced, and this contribution suffers from low recovery under dark conditions, even for long recovery times (~ days). The mechanism responsible for this semi-permanent V_th_ degradation (M2_V_HIGH_ in Figure 14) is due to the worsening of M2 under high fields, resulting in electron transport from the channel to energetically deeper trap states along the interface, or further within the bulk of the dielectric. To enable de-trapping from these deeper trap states, light energy ≥2.9 eV is required.

## 5. Conclusions

In this paper, we have summarized some of the most relevant challenges for the development of reliable GaN-on-Si vertical trench MOSFETs, for application in power electronics. Specifically, we presented the results of recent case studies, aimed at investigating (a) the origin of OFF-state leakage current, (b) the role of p-body doping in determining the breakdown voltage of the vertical stack, (c) the substantial improvement of reliability that can be obtained through the use of a bi-layer gate insulator, (d) specific failure mechanisms related to the optimization of the trench etching and cleaning procedure, and (e) a set of advanced results on the physics of interface trapping phenomena, obtained through the use of pulsed/transient measurements carried out in dark and under light. The obtained insights help understanding the current issues faced by the GaN for power community, and demonstrates strategies for identifying and analyzing the structural, leakage and trapping constraints to realize efficient and economical GaN-on-Si devices. If the pace of development and innovation within GaN-on-Si technologies is sustained, the benefits could prove to be revolutionary for the power semiconductor industry.

## Data Availability

No new data were created or analyzed in this study.
